# An improved seam carving method for enhancing the visual field of tunnel vision patients

**DOI:** 10.1038/s41598-026-35527-0

**Published:** 2026-02-03

**Authors:** Dina El-Torky, Salsabil El-Regaily, Ahmad Moadamani, Ahmed Osama, Alaa Mostafa, Amira Yasser, Mosaab Ghaley, Shahd Ashraf, Maryam Al-Berry, Zaki Fayed

**Affiliations:** 1 Basic Sciences Department, Faculty of Computer and Information Sciences, Ain Shams University, Cairo, Egypt; 2 Scientific Computing Department, Faculty of Computer and Information Sciences, Ain Shams University, Cairo, Egypt; 3 Computer Science Department, Faculty of Computer and Information Sciences, Ain Shams University, Cairo, Egypt

**Keywords:** Content-aware image retargeting, Seam carving, Visual field, Visual aid, Computer science, Software, Energy science and technology, Mathematics and computing

## Abstract

Visual impairment has various forms all of which negatively affect the patient’s daily activities and prevent performing simple actions like walking safely in a street. Content-aware image retargeting can be used to enhance the scene for patients who have limited visual field i.e. tunnel vision. A modified Seam Carving method is presented in this research paper which can decrease the width of the input image to fit in the patient’s angle of vision while preserving the important objects in the original image as well as the image details. The method enhanced the original Seam Carving by calculating the energy map using multiscale image fusion that combines depth, saliency, foreground segmentation, and edge detection features, and used a forward-middle approach for the seam removal step. The results showed efficiency that outperformed various retargeting methods, achieving a 30.8% improvement in the composite score that integrates structural, perceptual, and feature-based quality metrics. Statistical analysis using paired t-tests ($n = 73$) confirmed statistically significant improvements across all major metrics ($p<0.001$), including SSIM, SIFT feature matching, and modern deep learning-based perceptual quality metrics, compared to the baseline seam carving method.

## Introduction

With the rapid development of display devices, the role of Content Aware Image Retargeting (CAIR) is increasing, as it aims to adjust the dimensions of the displayed image on different output devices with various dimensions keeping all the image features and preserving its quality. On the other hand, CAIR methods can be very effective if used with visual aids to compress the images width to fit the limited visual field of visually impaired people. Visual field is the area that can be seen while the person is fixing their eyes straightly forward^1^. The loss of visual field can be caused by various diseases such as glaucoma, retinitis pigmentosa, and some types of brain tumors. This can significantly impact the quality of life of the patients decreasing their ability to perform their daily activities, as well as increasing the risk of falling, car accidents and even mortality^2^.

Image retargeting emerges as a promising approach to enhance visual accessibility for individuals with visual impairments by modifying image characteristics to match their visual capabilities. One of the important trials in this field is the method proposed by Atabani et al.^3^, which combined shrinkability and seam carving methods to decrease the width of images without changing the size of the important objects. The method produced efficient results, however, the results were only compared with the shrinkability and seam carving methods.

This paper presents a novel image retargeting algorithm that makes the image more suitable for patients with limited visual fields while preserving all important objects in the scene as well as the quality of the output image.

The rest of the paper is organized as follows; “Related work” reviews the related work in the field, highlighting key contributions to content-aware image retargeting. In “Proposed approach”, we present the proposed approach, detailing each step of the methodology. Section “Results” showcases the experimental results and evaluates the method’s performance using established quality metrics. Finally, “Conclusion” concludes the paper by summarizing the research findings and discussing potential future directions.

## Related work

Earlier, traditional image retargeting techniques were introduced to change the images dimensions such as fixed cropping and uniform scaling. These methods either changed the aspect ratio of the objects causing distortions or eliminated objects from the image without considering their importance. As a result, CAIR methods were introduced to overcome the drawbacks of the traditional methods^4^.

Abundant CAIR techniques were introduced and classified into different types such as discrete, continuous and multi-operator methods. The discrete methods include content-aware cropping, shift map and seam carving-based methods, while the continuous methods enclose content-aware scaling and warping-based methods. On the other side, multi-operator methods combine two or more retargeting methods trying to overcome the drawback of using any of the CAIR methods solely. Lastly, since Deep Learning (DL) has become a crucial approach in solving any emerging problem, various DL-based methods were proposed to solve the image retargeting problem^5–7^. Recent work by Givkashi et al.^8^ demonstrated that supervised deep learning approaches can learn content-aware retargeting directly from data. While these methods show promise, they require large training datasets and may not generalize well to the specific visual characteristics important for visually impaired assistance (e.g., preserving small obstacles, maintaining spatial relationships for navigation).

Content-aware cropping methods try to adjust the cropping window so as it fits as many important objects as possible inside it. These methods mainly vary in the way of calculating the region of interest (ROI). Cavalcanti et al.^9^ used a genetic algorithm while^10^ used Particle Swarm Optimization to calculate the ROI. Su et al.^11^ proposed a Spatial-Semantic Collaborative cropping network. The main advantage of Content-aware cropping methods is that they preserve the image structure, but on the other side, they fail in case of having multiple ROI^5^.

Seam Carving (SC) can be considered as one of the most popular CAIR methods, it was introduced by Avidan and Shamir^12^. The method identifies and removes seams, or paths of least importance, from an image, preserving significant features while resizing. Since the original “backward” seam carving method calculated the energy of each pixel and found the optimal seam to remove based on this energy map, distortions were formed in the output image because new edges were formed after removing seams that were not adjacent in the original image. As a result, a forward SC approach was proposed by Rubinstein et al.^13^ who implemented a look-ahead mechanism that considered the impact of removing a seam on the rest of the image. Shen et al.^14^ proposed an algorithm that used content-aware seam-carving for preliminary retargeting then applied an adaptive repainting module to improve the quality of the output image.

Shift map method identifies similar patterns and removes them from the consistent parts of the image. An optimization problem of graph labeling is used where the shift map represents the label of each output pixel^15^. This method is not efficient with images containing large or multitudinous objects.

Content-aware scaling is a continuous CAIR method that designates different scaling factors to each image region according to its importance. Various techniques were proposed depending on this idea like^16–18^ and^19^. The usage of different scaling factors in these methods may cause some distortion at the boundaries separating important and unimportant regions.

Warping is also a continuous CAIR method that is based on distortion of image regions according to their importance, where important regions undergo minimum distortion while maximum distortion is applied to less important regions. Various techniques were proposed based on warping such as^20–22^ and^23^. In comparison to other CAIR methods, warping-based methods consume less execution time and conserve the image information more efficiently. On the other side, some details of the output image may be lost due to interpolation.

Combining two or more of the previously-mentioned CAIR methods is named multi-operator method. This was applied to overcome the drawbacks caused by using any of the CAIR methods separately. Numerous techniques were introduced combining different methods as in^24^ that combined weighted seam carving with grid-based warping. Also the researchers in^25^ introduced a technique called SCAN which combined seam carving, cropping, adding seams and normalization. More multi-operator methods are being proposed continuously aiming to improve the performance of CAIR using the combination of different existing CAIR methods.

## Proposed approach

**Figure 1:**
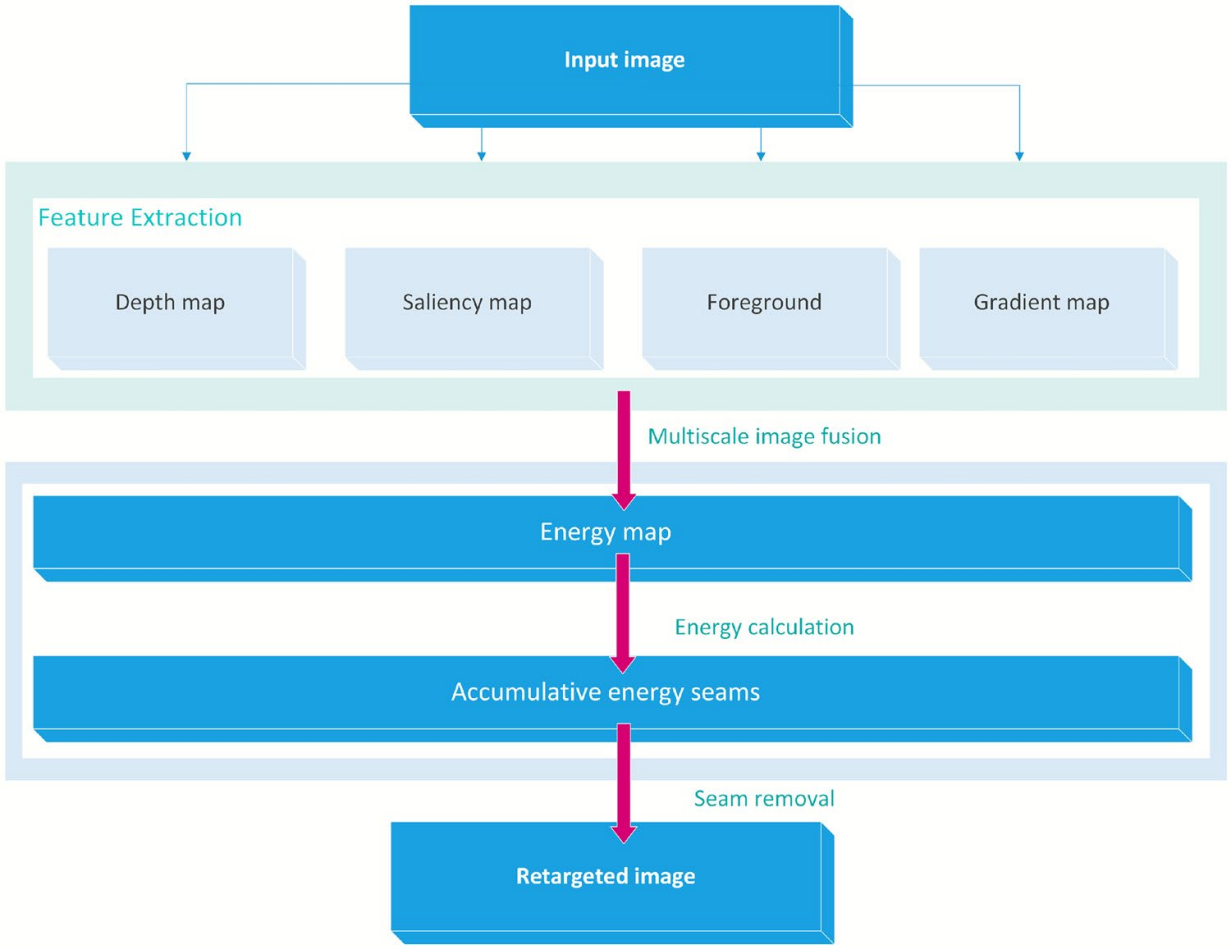
System architecture of the proposed approach.

The proposed method relies on the well-known Seam Carving method which is widely used for image retargeting. As mentioned in the previous section, SC involved calculating the energy map of the image and considered the seam as an 8-connected path of pixels. The least energy seam was removed and the process continued until the retargeted image reached the desired width. Our approach comprises three major steps which are Feature Extraction, Energy map Generation and Seam Removal. The technique involves calculating the feature map using a multiscale image fusion approach, where four different feature maps are combined to produce a comprehensive map that integrates all critical features from every perspective. The method also introduces a modified forward-middle approach for energy calculation instead of the top-down or bottom-up known approaches. Figure 1 demonstrates the system architecture of the proposed approach.

### Feature extraction

When dealing with a specific type of image content like faces, medical images, indoor/outdoor scenes or even text, the process of feature extraction can be simple and easily implemented. However, retargeting images for visually impaired people involves dealing with all types of image content, therefore, extracting image features should be more complicated. Our method uses four different approaches to calculate the image features producing four different maps for the input image.

#### Depth estimation

A Depth map represents a scene by encoding the distance of each pixel from the viewing point, thereby providing spatial information about the scene. This can help giving a higher priority to the foreground objects thus reducing the loss of important objects from the retargeted image. MiDaS v3.11 for monocular depth estimation^26^ was used to produce a depth map for the input image. MiDas is a deep learning-based model that is based on a CNN with an encoder-decoder structure where the encoder is a pre-trained convolutional network which extracts features at multiple scales, while the decoder processes these features to produce the required depth map. This method is robust since it is trained on diverse datasets, also it is fast and efficient to be used with a real-time system.

#### Saliency detection

Saliency detection aims to identify the objects or regions in the image that are distinct from their surroundings hence more likely to attract the attention of the observer. U^2^-Net^27^ was used for Salient Object Detection (SOD).

U^2^-Net differs from the design of most existing SOD networks that depend on features extracted by existing backbones, which are basically designed for the purpose of image classification. They focus on features that represent the semantic meaning apart from local details and global contrast information, both of which are crucial for saliency detection. U2-Net is a two-level nested U-structure that is designed not to rely on any pre-trained backbones from image classification. In addition, the network architecture helps attaining high resolution without increasing the computational cost^27^. This is achieved by the nested structure where on the bottom level, a ReSidual U-block (RSU) is designed, which is able to extract intra-stage multi-scale features without affecting the resolution of the feature map. A U-Net like structure exists on the top level, where each stage is filled by an RSU block.

#### Foreground segmentation

Foreground segmentation separates the main image objects from the background. It is invaluable for protecting the integrity of the main subjects by giving them more weight than the background. In addition, it may occasionally solve the saliency failure cases, allowing for more processing of background areas^28^.

To implement foreground segmentation, we used Mask R-CNN^29^ along with the depth map resulted from the MiDas model. Mask R-CNN is a faster version of the region-based convolutional neural network (R-CNN) that is used for object instance segmentation^30^.

First, the output objects of the Mask R-CNN are filtered out keeping objects with confidence higher than 0.7. Then, for each bounding box, the average depth value is calculated using the depth map produced from MiDas model. Finally, the masks are filtered based on the average depth values where values above 0.7 are selected. The confidence threshold of 0.7 for Mask R-CNN was selected based on the model’s precision-recall trade-off curve, where values below 0.7 included excessive false positives while values above 0.7 showed diminishing returns. Similarly, the depth threshold of 0.7 was chosen to focus on foreground objects, as MiDaS outputs normalized depth values where higher values indicate closer objects.

#### Edge detection

The structural integrity and details of the image cannot be conserved without detecting the edges of the image. Sobel filter^31^ was used to detect the vertical and horizontal edges in the image. This filter calculates the image gradient by applying convolution with two special masks to calculate the vertical and horizontal edges of the image.

### Energy map generation

The idea of the used energy map generation method was inspired from^32^ where the four maps that were produced from the feature extraction step and explained in the previous section were used to calculate the combined energy map, which is the essential input for Seam Carving. Multi-scale Image Fusion was used to combine these maps and create the energy map. Multi-scale fusion is a technique that is used to combine multiple images, enhancing the final output, by integrating data at different scales. This allows capturing the fine details in small scales while maintaining structure in large ones.

**Figure 2:**
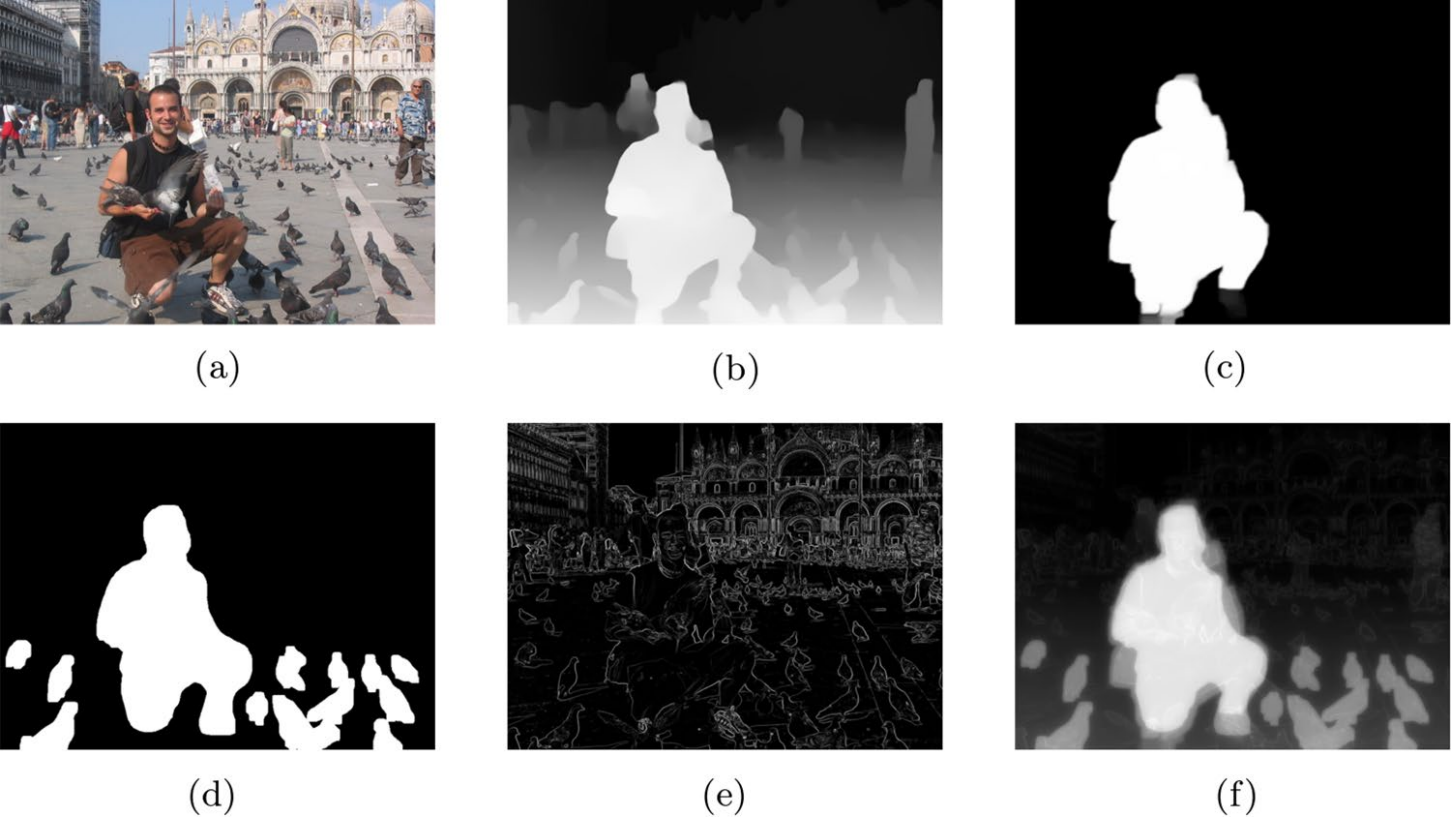
Feature extraction steps to form energy map.

Five Gaussian pyramid levels were used to decompose the images into different scales. Different weights were assigned to each feature map. At each scale, fusion rules were applied to combine the four feature maps. The weights [0.3, 0.3, 0.3, 0.1] for depth, saliency, gradient, and foreground respectively were determined through empirical evaluation on 20 validation images. We tested weight combinations in 0.1 increments and selected the configuration that maximized the average SSIM and SIFT scores. Finally, the final fused image was reconstructed from the combined multi-scale images. Conflicts can arise when different maps provide contradictory information–for example, an important background object (represents high saliency) that is distant (low depth). Our multi-scale fusion approach prevents these conflicts by operating at multiple scales, allowing fine details and broad structure to be captured separately. The following pseudocode 1 explains the steps of applying multi-scale image fusion. Figure 2 illustrates the steps of extracting the four different feature maps from the original image and combining them to form the final energy map.

**Algorithm 1:**
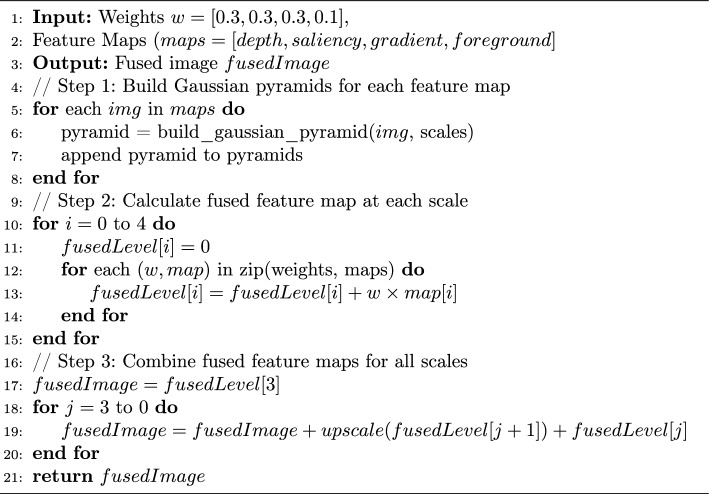
MultiscaleFusion

### Energy calculation and seam removal

The goal of this step is to choose the least important seams to be removed while ensuring not to affect the structure of the output image or cause distortion. The first step is to compute the cumulative energy map $M(x,y)$ in a top-down manner using the energy map $E(x,y)$ as a reference. This is done by memorizing the cumulative energy for each pixel, where the value at each pixel represents the total energy of the least energy path leading to that pixel.

Backward energy accumulation is the method used in the original seam carving algorithm to determine the optimal seams for removal. It calculates the cumulative energy for each pixel by comparing it to the energy of pixels in the previous row (i.e., the row above). Specifically, for each pixel $(x,y)$, the cumulative energy $M(x,y)$ is determined by adding the pixel’s energy $E(x,y)$ to the minimum cumulative energy of the three possible preceding pixels: upper left $M(x-1,y-1)$, upper middle $M(x,y-1)$, and upper right $M(x+1,y-1)$.

This approach finds the shortest path from the top of the image to the bottom, ensuring that the least important seams are identified for removal. This method produced artifacts in the image because it didn’t consider the impact of removing a seam on its neighboring pixels.

On the other hand, forward energy accumulation is another method used in seam carving that evaluates the seam removal more comprehensively^13^. Instead of only looking at the previous row, this method also considers the future impact of removing a pixel. For each pixel $(x,y)$, the algorithm calculates the forward energy $F(x,y)$ by adding the pixel’s energy $E(x,y)$ to the minimum cumulative energy of the three possible pixels: upper left $F(x-1,y+1)$, upper middle $F(x,y+1)$, and upper right $F(x+1,y+1)$. This approach takes into account how removing certain pixels would affect the surrounding image structure.

This calculation can be expressed as $F(x,y) = min(CL,CU,CR)$ where CL, CU and CR are the cumulative energy of the three possible neighboring pixels which are computed according to each case as shown in equation 1:

1 $$\begin{aligned} \begin{aligned}&CL = |I(x,y-1)-I(x,y+1)| + |I(x-1,y)-I(x,y-1)| \\&CU = |I(x,y-1)-I(x,y+1)| \\&CR = |I(x,y-1)-I(x,y+1)| + |I(x-1,y)-I(x,y+1)| \end{aligned} \end{aligned}$$

This approach helped ensure that the selected seams for removal maintain the image’s structural integrity and visual coherence, providing more balanced and aesthetically pleasing results. The seam removal step involves following a bottom-up approach to remove the calculated seams based on the cumulative energy map generated in the previous step. Some methods use the masking approach for seam removal. This approach involves marking the pixels to be removed with a unique value for easy identification later. After every three cycles, the marked pixels are removed from each row. The iterative approach is more straightforward. Starting from the bottom and moving up, each unnecessary pixel in the identified seam is removed, and the original image is replaced with a new image that is one pixel narrower. This process is repeated until the desired width is achieved.

Initial attempts to partition the image into multiple vertical columns proved inefficient due to inter-column dependencies requiring excessive synchronization overhead, motivating our horizontal partitioning strategy. The presented forward-middle approach opted to split the image horizontally into two equal halves, allowing each half to be processed independently. The energy accumulation for the top half worked from top to bottom, while the bottom half worked from bottom to top, converging in the middle. Using the middle row, we identified the least energy pixel as the starting point in both directions. Then, we began removing seams from bottom to top in the upper half and from top to bottom in the lower half. This method produced a connected seam that met in the middle. The seam now had more spatial range to traverse, which was particularly beneficial when the best seam was diagonal.

The forward-middle approach is motivated by two key observations. First, from a perceptual point where the most visually salient information and the natural center of visual attention typically exist in the middle of an image rather than at the top or bottom edges. By initiating energy accumulation from the middle row and propagating bidirectionally (upward and downward simultaneously), we ensure that the energy calculation prioritizes the most important region first. Second, this approach provides computational benefits for error propagation. In traditional top-to-bottom energy accumulation, quantization errors accumulate across the entire image height. By splitting the accumulation into two independent paths from the middle, we effectively reduce the maximum error propagation distance by half. Each path accumulates errors over *h*/2 rows instead of *h* rows, where *h* is the image height.

The method was implemented in Python and tested using 73 images from the *RetargetMe* dataset^33^. It was specially designed to test image retargeting using different methods. It consists of 91 images with different dimensions and various properties.

The forward-middle approach achieves $O(n\times m/2)$ complexity for each half processed in parallel, where *n*, *m* represent image dimensions. This results in a theoretical speedup factor of 2 compared to the sequential $O(n\times m)$ approach, though practical speedup depends on parallelization overhead and hardware capabilities. The efficiency of the proposed method is illustrated in the following section.

## Results

**Figure 3:**
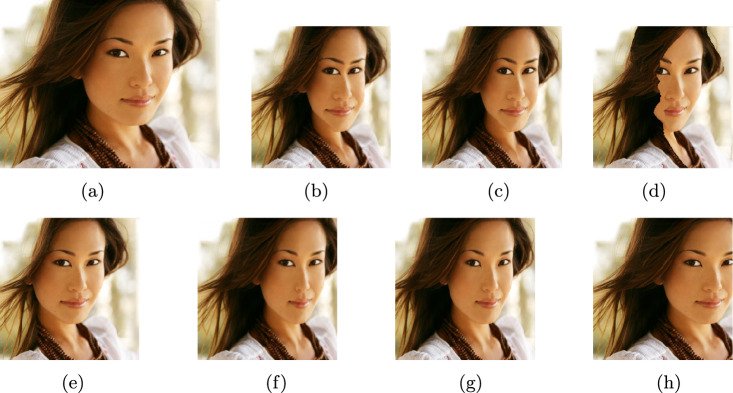
Image retargeting results.

In this section, the proposed technique is compared with six different image retargeting techniques. Figure 3 illustrates a sample of the output retargeted images produced by each method examined in this study. The first is the original Seam Carving method^12^ which used the gradient map as the energy map and calculated the accumulative seam energy using backward method. The second is the modified Seam Carving method^13^ that used the forward energy calculation method, while the third is also a modified Seam Carving method^34^ that used the forward energy calculation method and the saliency to calculate the energy map. In addition to Shift Map, Warping and Multi-operator methods that were used for comparison in the RetargetMe dataset^33^.

Examining the sample results in Fig. 3, our method demonstrates superior performance in several aspects. The output (h) preserves the structural image features which is the mostly required for visual impairment assistance unlike images (b),(c) and (d). Additionally, the proposed method’s output produced the least distortion as shown, in contrast to images (d) and (e). Finally, the aspect ratio of the objects in the retargeted images (f) and (g) are clearly affected whereas the proposed method shows a significant aspect ratio preservation.

Seven quality metrics were calculated for each method. The first and second are Structural Similarity Index Measure (SSIM) and Peak Signal to Noise Ratio (PSNR) where higher values indicate better quality of the output image^35^. In the third metric, the Scale-Invariant Feature Transform (SIFT) identifies distinctive keypoints in the source and re-targeted images, then a matching operation is performed between these keypoints to calculate the similarity score^36^. Higher values of SIFT match also indicate better retargeted image. Euclidean distance of Histogram (EH), which measures the distance between histograms of the original and the output images indicates higher similarity for smaller distance. The last two metrics are Learned Perceptual Image Patch Similarity (LPIPS)^37^ and Deep Learning-based quality metric (DL) proposed by Absetan et al.^38^. Finally, a composite score (CScore) is proposed and calculated to combine all the measured quality metrics using equation 2. Each quality metric was assigned a certain weight according to its importance.

2 $$\begin{aligned} \begin{aligned} CScore&= 0.30 \times SSIM + 0.25 \times SIFT + 0.20 \times DL \\&+ 0.10 \times PSNR + 0.10 \times LPIPS + 0.05 \times (1 - EH) \end{aligned} \end{aligned}$$

The composite score (CScore) was calculated to provide a single quality measure integrating all evaluated metrics. The weights were assigned based on the relative importance of different aspects for visual assistance applications: structural preservation (SSIM, 0.30) receives the highest weight as it is critical for spatial understanding and navigation safety; feature preservation (SIFT, 0.25) is weighted second to ensure recognizing objects and landmarks; modern perceptual quality (DL, 0.20) captures deep learning-based quality assessment; pixel-level accuracy (PSNR, 0.10) and perceptual similarity (LPIPS, 0.10) provide complementary measures; while color consistency (EH, 0.05) receives minimal weight as it is less critical for obstacle avoidance and navigation tasks. Table 1 shows the quality metrics values for the mentioned CAIR methods. These metrics were calculated for each image in the dataset, then an average score was calculated to give a single value for each quality metric.

**Table 1:** Quality metrics values for different CAIR methods.

Method/quality metric	SSIM	PSNR	SIFT	EH	LPIPS	Deep L.	CScore
Original SC^12^	0.34	11.74	70.2	0.014	0.326	0.395	19.10
Modified SC (fwd)^13^	0.37	11.72	72.3	0.020	0.3315	0.694	19.63
Modified SC (fwd/Saliency)^34^	0.56	13.76	88.6	0.014	0.335	0.737	23.97
Shift map^33^	0.46	12.35	67.9	0.022	0.468	0.74	18.64
Warping^33^	0.43	11.31	48.2	0.056	0.274	0.674	13.57
Multi-operator^33^	0.51	10.93	47.1	0.058	0.253	0.644	13.27
**Proposed method**	**0.58**	**14.77**	**92.1**	**0.010**	**0.338**	**0.741**	**24.97**

### Ablation study

To evaluate the individual contributions of our two main contributions, we conducted an ablation study comparing baseline backward seam carving with multi-feature energy map using backward seam removal, forward-middle approach with gradient energy map, and finally, the full proposed method combining both innovations. Table 2 shows that the multi-feature energy map provides the largest quality improvement, increasing CScore from 19.05 to 24.23. This shows that the systematic integration of depth, saliency, foreground, and edge information significantly enhances feature preservation. The forward-middle seam removal approach contributes an additional improvement when used alone. The full model achieves a CScore of 24.52, showing that both components integrate to produce the best overall quality.

**Table 2:** Ablation study: component contribution analysis.

Configuration	SSIM	PSNR	EH	SIFT	DeepJSD	LPIPS	CScore
Baseline (backward SC)	0.334	13.79	0.012	88.15	0.696	0.326	19.05
+ Multi-feature energy	0.430	13.91	0.016	88.89	0.742	0.321	24.23
+ Forward-middle	0.411	13.75	0.011	89.89	0.728	0.341	23.07
**Full model (proposed)**	**0.574**	**14.55**	**0.016**	**90.63**	**0.741**	**0.339**	**24.52**

### Computational performance

The runtime comparison of different seam carving configurations on 73 test images is presented in Table 3 . All experiments were conducted on a system with Intel Core i5 processor running at 5.0GHz, 16GB RAM, and NVIDIA GeForce GTX 1650 (4GB VRAM) under Linux 6.14.0-36-generic. The proposed method achieves a mean runtime of 2.54 seconds per image, which represents a 21.5% increase compared to the baseline backward seam carving (2.09 seconds). This moderate overhead is primarily caused by the multi-feature energy map calculation, which includes depth estimation (MiDaS), saliency detection ($\text {U}^{2}$-Net), foreground segmentation (Mask R-CNN), and multi-scale fusion. In addition, the forward approach consumes more execution time than the backward approach due to its more complex processing steps. The relatively high standard deviations (1.16-1.41 seconds) across all methods reflect the natural variation in processing time due to different image sizes and content complexity in the dataset. The minimum runtime (0.55-0.67 seconds) corresponds to smaller images with simpler content, while maximum runtime (3.85-4.79 seconds) corresponds to larger, more complex images requiring more seams to be removed. These results demonstrate that the proposed method achieves substantial quality improvements (30.8% CScore enhancement) with a moderate computational cost increase (21.5%).

**Table 3:** Runtime comparison of seam carving methods (seconds).

Statistic	Baseline (backward SC)	Multi-feature	Forward-middle	Proposed
Mean	2.09	2.41	2.05	2.54
Min	0.55	0.59	0.55	0.67
Max	3.93	4.55	3.85	4.79
Std Dev	1.17	1.35	1.16	1.41

### Statistical significance

Statistical analysis using paired t-tests confirmed that the proposed method achieved statistically significant improvements over the original Seam Carving method across 7 out of 8 quality metrics $(n=73, \alpha =0.001)$ as shown in Table 4. The proposed method demonstrated a substantial improvement in structural similarity $(SSIM: 0.574 \ vs. 0.334,\ t(72)=13.82,\ p<0.001)$, indicating superior preservation of image structure critical for navigation tasks. Feature preservation, measured by SIFT keypoint matching, showed the most dramatic improvement $(92.2\% \ vs. 70.2\%,\ t(72)=17.41,\ p<0.001)$, suggesting that distinctive objects and landmarks remain more recognizable after retargeting. The deep learning-based quality metric by Absetan et.al^38^ also showed highly significant improvement $(0.741 \ vs. 0.696,\ t(72)=13.87,\ p<0.001)$, confirming superior perceptual quality from a deep learning perspective. LPIPS^37^, another perceptual similarity metric, demonstrated significant improvement $(0.405 \ vs. 0.326,\ t(72)=7.09,\ p<0.001)$. The composite score (CScore), which integrates all quality dimensions with application-specific weights, improved dramatically from 19.05 to 24.93 $(t(72)=17.39,\ p<0.001)$, representing a 30.8% overall quality enhancement. Peak signal-to-noise ratio (PSNR) also showed significant improvement $(14.8 \ vs. 11.7,\ t(72)=3.89,\ p<0.001)$. Only the Euclidean histogram distance (EH) showed no significant difference $(p=0.773)$, indicating comparable color distribution preservation. These results provide overwhelming statistical evidence that the improvements are genuine and not due to random variation.

**Table 4:** Statistical significance test: proposed method vs. original SC.

Metric	Proposed	Original SC	t-statistic	p-value	Significance
SSIM	0.5741	0.3340	13.82	<0.001	***
PSNR	14.77	11.74	3.89	<0.001	***
SIFT	92.17	70.24	17.41	<0.001	***
LPIPS	0.4045	0.3264	7.09	<0.001	***
DeepJSD	0.7412	0.6956	13.87	<0.001	***
EH	0.0148	0.0144	0.29	0.773	ns
CScore	24.93	19.05	17.39	<0.001	***

Note: Paired t-test, n=73.

*** indicates p< 0.001 (highly significant), ns = not significant.

## Conclusion

In this paper, we presented a modified Seam Carving technique for image retargeting aiming to enhance the scene for patients with peripheral vision loss. The method performed content-aware image retargeting to decrease the width of the image so it can fit into the patient’s visual field. First, a feature extraction step was performed to calculate four different maps (Depth map, Saliency map, Segmented Foreground and Gradient map) which are combined together using Multiscale image fusion to form an Energy map. Next, energy calculation step is applied to decide the less important seams that need to be removed using a forward-middle approach. Finally, seam removal is implemented on each half of the image simultaneously.

RetargetMe dataset was used to apply the proposed method. The output images preserved the important objects while maintaining the image details producing less distortion. Seven quality metrics were calculated for the proposed method and compared with six image retargeting methods. The proposed method showed superiority over the other methods in six out of seven metrics, achieving a 30.8% improvement in the composite score compared to the baseline seam carving method. Statistical analysis confirmed statistically significant improvements (p< 0.001) across structural similarity, SIFT feature preservation, and deep learning-based quality metrics. An ablation study revealed that the multi-feature energy map provides the primary quality contribution (+5.18 CScore points), while the forward-middle approach adds further gains (+4.02 points).

Future work will address three key technical challenges: Temporal coherence for video retargeting through seam consistency across frames; Patient-specific customization by adapting feature weights to individual visual field profiles; Clinical validation with tunnel vision patients measuring task performance metrics including obstacle avoidance accuracy and navigation time in controlled environments.

## Data Availability

The RetargetMe dataset is publicly available at https://people.csail.mit.edu/mrub/retargetme/.
